# The Role of Genetic Risk Factors in Pathogenesis of Childhood-Onset Systemic Lupus Erythematosus

**DOI:** 10.3390/cimb45070378

**Published:** 2023-07-17

**Authors:** Mario Sestan, Nastasia Kifer, Todor Arsov, Matthew Cook, Julia Ellyard, Carola G. Vinuesa, Marija Jelusic

**Affiliations:** 1 Department of Paediatrics, University of Zagreb School of Medicine, University Hospital Centre Zagreb, 10000 Zagreb, Croatia; 2 Faculty of Medical Sciences, University Goce Delchev, 2000 Shtip, North Macedonia; 3 The Francis Crick Institute, London NW1 1AT, UK; 4 Department of Immunology and Infectious Diseases, The John Curtin School of Medical Research, Australian National University, Canberra, ACT 2601, Australia; 5 Department of Medicine, University of Cambridge, Cambridge CB2 1TN, UK

**Keywords:** childhood-onset systemic lupus erythematosus, monogenic systemic lupus erythematosus, genetics, pathogenesis

## Abstract

The pathogenesis of childhood-onset systemic lupus erythematosus (cSLE) is complex and not fully understood. It involves three key factors: genetic risk factors, epigenetic mechanisms, and environmental triggers. Genetic factors play a significant role in the development of the disease, particularly in younger individuals. While cSLE has traditionally been considered a polygenic disease, it is now recognized that in rare cases, a single gene mutation can lead to the disease. Although these cases are uncommon, they provide valuable insights into the disease mechanism, enhance our understanding of pathogenesis and immune tolerance, and facilitate the development of targeted treatment strategies. This review aims to provide a comprehensive overview of both monogenic and polygenic SLE, emphasizing the implications of specific genes in disease pathogenesis. By conducting a thorough analysis of the genetic factors involved in SLE, we can improve our understanding of the underlying mechanisms of the disease. Furthermore, this knowledge may contribute to the identification of effective biomarkers and the selection of appropriate therapies for individuals with SLE.

## 1. Introduction

Systemic lupus erythematosus (SLE) is a chronic autoimmune condition with the potential to affect any organ system. It is characterized by the presence of antibodies that specifically target nuclear and cytoplasmic antigens, leading to the widespread inflammation of blood vessels and connective tissue [1]. This immune response also triggers complement activation and the deposition of immune complexes. Consequently, SLE is commonly referred to as “the disease with countless manifestations” due to its capacity to involve multiple organs and exhibit a wide range of clinical symptoms, varying from mild to life-threatening. The inflammatory process typically impacts the skin, kidneys, brain, lungs, and heart [2].

Around 15–20% of individuals with SLE experience the onset of the disease during childhood and receive a diagnosis before the age of 18 [3,4]. This specific form is commonly referred to as cSLE. Although there are similarities in the clinical presentation and immunological markers between children and adults with SLE, it is crucial to recognize cSLE as a distinct clinical entity due to several unique characteristics.

There are significant variations in disease manifestations between these two age groups [3,5,6,7] (Table 1).

Firstly, in childhood, the clinical picture at the time of diagnosis tends to be more severe, with symptoms such as proteinuria, hemolytic anemia, leukopenia, and a rash in the zygomatic region [3,5,8]. Additionally, cSLE often affects multiple organs and systems, with a predilection for kidney involvement. Renal complications occur in approximately 60–80% of children and 35–50% of adults, as suggested by the literature [5]. Furthermore, there is a notable disparity in central nervous system involvement, affecting 20–50% of children and 10–25% of adults [5]. Conversely, lung issues (20–90% in adults compared to 15–40% in children) and joint problems (80–95% in adults compared to 60–70% in children) are more frequently observed in adult patients [9,11].

In cSLE, procedures like renal biopsies, dialysis, and transplantation are more common, while convulsions occur more frequently and the risk of myocardial infarction is elevated. Generally, cSLE follows a more aggressive clinical course, increasing the likelihood of permanent organ and system damage over time. Considering these factors, children require more intensive treatment, often involving the use of glucocorticoids and immunosuppressants. Consequently, compared to adults, children face a significantly higher risk of corticosteroid-related complications, such as cataracts and avascular bone necrosis [12].

Unlike SLE in adults, where the disease is approximately nine times more prevalent in females, the gender difference in children with cSLE is significantly less pronounced, with a ratio of approximately 4–5 girls to 1 boy. Additionally, it is important to note that cSLE is associated with primary immunodeficiencies, particularly deficiencies in complement components, which contribute to a higher disease activity index. Moreover, when considering early-onset SLE in children, genetic factors may play a more significant role compared to environmental and hormonal factors, which differs from adult-onset SLE [8]. Consequently, rare monogenic forms of SLE resulting from mutations in specific genes occur more frequently in childhood SLE. These monogenic forms follow Mendelian inheritance patterns and have fundamentally challenged the previously established notion of SLE as a solely polygenic disease [13]. Additionally, certain variants exhibited distinguishable differences between cSLE and SLE [10].

This review aims to provide a comprehensive overview of the current understanding of monogenic and polygenic SLE, focusing on the implications of specific genes in disease pathogenesis. By conducting an in-depth analysis of the genetic factors contributing to SLE, we can enhance our comprehension of the underlying disease mechanisms. Furthermore, this knowledge may assist in the identification of effective biomarkers and aid in the selection of appropriate therapies for individuals with SLE.

## 2. Polygenic SLE

The precise causes of cSLE are complex and not yet fully understood. The etiology of cSLE involves three primary factors: genetic risk factors, epigenetic mechanisms, and environmental triggers [14].

Genetic factors play a significant role in the development of the disease. In the general population, the risk of developing SLE is approximately 0.1%, while for females, it is around 0.2%. On average, about 7% of SLE patients have first-degree relatives with the same disease [15]. The risk for first-degree relatives ranges from 4% to 8% [16], but in some cases, it can be higher, with sisters of SLE patients having a risk of up to 10% [17]. In countries where consanguineous marriages are more prevalent, the risk can be significantly higher. Siblings of SLE patients have an 8 to 20 times higher risk of developing the disease compared to the general population [18,19,20]. The strong influence of genetics is evident from the fact that monozygotic twins have a 10-fold increased risk compared to dizygotic twins [19,21]. The estimated heritability of SLE ranges from 44% to 66%, with a concordance rate of approximately 24% to 56% among monozygotic twins, whereas in dizygotic twins, it is only 2% to 5% [22,23,24,25,26].

The presence of autoimmune diseases within the family poses a risk factor for the development of SLE, and this risk escalates with the number of relatives affected by autoimmune conditions. Genome-wide association studies (GWAS) have identified over 100 gene loci associated with susceptibility to SLE, although these loci may also contribute to the development of other autoimmune diseases [27,28]. Consequently, having a family history of autoimmune disease increases the risk of SLE by a factor of 4.1, and this risk further rises with the number of relatives affected by autoimmune disease, reaching up to 11.3 times higher [29].

**Figure 1:**
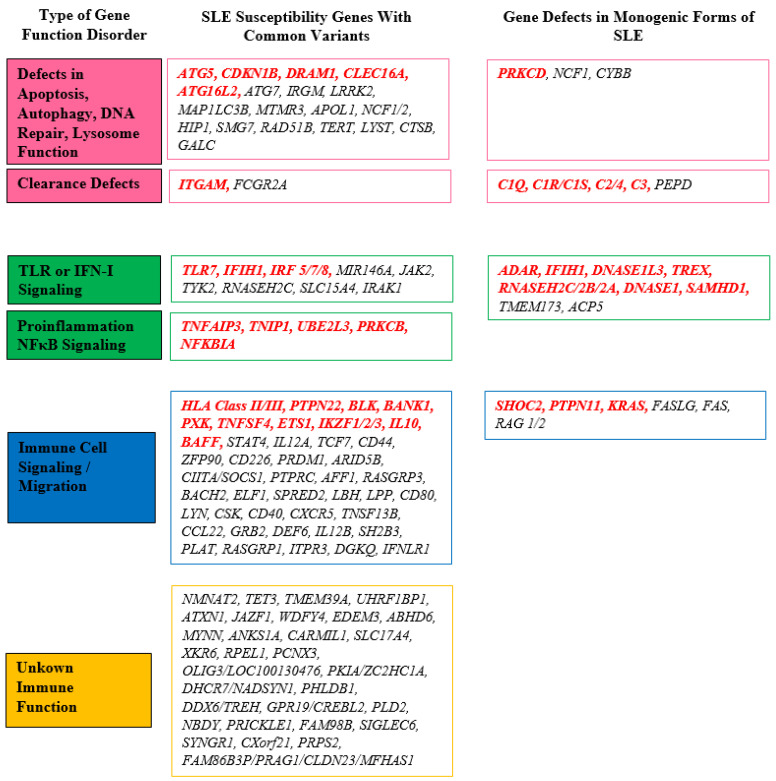
Overview of the important genes involved in SLE pathogenesis. The most important genes are marked in red. Modified according to reference [26]. DNA: deoxyribonucleic acid; IFN-I: type I interferon; NFκB: nuclear factor κB; SLE: systemic lupus erythematosus; TLR: Toll-like receptor.

The initial gene association identified in SLE was the major histocompatibility complex (MHC) located on chromosome 6, which encompasses human lymphocyte antigens (HLA) [30]. Current knowledge categorizes SLE susceptibility genes into four groups [27] (Figure 1).

### 2.1. Genes Related to Apoptosis, Autophagy, DNA Repair, Lysosome Function, and Clearance of Immune Complexes

The initial group consists of genes involved in various processes such as apoptosis, autophagy, DNA repair, lysosome function, and immune complex clearance. These genes are categorized together because they are associated with the dysfunctional mechanisms mentioned earlier, which can result in the increased exposure of nuclear autoantigens to the immune system and the deposition of immune complexes. These processes are crucial in initiating and sustaining the autoimmune response in lupus. Autophagy, for example, is a cellular process known as “self-digestion” responsible for breaking down long-lived proteins and cytoplasmic organelles [31]. Autophagy-related mechanisms play a role in regulating multiple immune responses, including antigen delivery to major histocompatibility complex (MHC) compartments, lymphocyte survival and homeostasis, and cytokine production. Through GWAS, several autophagy-related genes, namely *ATG5*, *CDKN1B*, *DRAM1*, *CLEC16A*, and *ATG16L2*, have been identified as potentially associated with SLE susceptibility [31]. Additionally, other susceptibility genes such as *ATG7*, *IRGM*, *LRRK2*, *MAP1LC3B*, *MTMR3*, and *APOL1* play significant roles within this signaling pathway [27,31].

Autophagy-related 5 (Atg5), encoded by the *ATG5* gene in humans, is a key protein involved in the formation of autophagic vesicles and is central to autophagy. However, Atg5 also has diverse functions, including mitochondrial quality control after oxidative damage, negative regulation of the innate antiviral immune response, lymphocyte development and proliferation, MHC II antigen presentation, adipocyte differentiation, and apoptosis [32]. While it is known that both common and rare variants of ATG5 are associated with SLE susceptibility, the precise mechanism by which *ATG5* contributes to lupus is not yet fully understood [31]. There are indications that *ATG5* may initiate the development of SLE by promoting cytokine imbalance or disrupting antigen presentation.

Cyclin-dependent kinase inhibitor 1b (Cdkn1b) is an enzyme inhibitor encoded by the *CDKN1B* gene. It functions as an unconventional tumor suppressor and plays various roles in regulating the cell cycle, cell proliferation, and differentiation [33]. Its importance in T lymphocyte development is particularly notable, as it is crucial for inducing T-cell tolerance and anergy. Mice deficient in the cyclin-dependent kinase inhibitor p27 exhibit mild lupus-like abnormalities characterized by a decreased number and activity of regulatory T-cells (Treg cells) [33].

The DNA damage-regulated autophagy modulator 1 (*DRAM1*) gene encodes a lysosomal membrane protein that is essential for initiating autophagy [31]. *DRAM1* expression is induced following DNA damage caused by UV irradiation, which provides a possible explanation for its involvement in the development of SLE [31,34]. It potentially serves as a connection between genetic factors associated with autophagy and environmental triggers.

The C-type lectin domain family 16 member a (Clec16a) protein regulates the selective degradation of mitochondria through autophagy and influences T-cell selection and reactivity in the thymic epithelium [31]*. CLEC16A* has been genetically linked to multiple autoimmune disorders, including multiple sclerosis, rheumatoid arthritis, Crohn’s disease, and SLE. The exact mechanism by which *CLEC16A* contributes to the development of SLE is not yet understood. However, the observed reduced expression of *CLEC16A* isoforms in SLE may lead to increased autophagic activities [35].

*ATG16L2* (autophagy-related 16 like 2) is a gene that participates in autophagy and has been suggested as a genetic locus associated with an increased risk of SLE. It has also been linked to multiple sclerosis and Crohn’s disease. While *ATG16L2* is believed to have a significant role in autophagy, especially in T-cells, the specific nature of its involvement is still unknown [36].

Among the genes related to SLE susceptibility, particularly in terms of immune complex clearance, the *ITGAM* gene stands out. The Integrin alpha M (*ITGAM*) gene encodes the integrin alpha M chain, which combines with the beta 2 chain to form either macrophage receptor 1 (Mac-1) or complement receptor 3 (CR3). These receptors play a crucial role in facilitating the adherence of neutrophils and monocytes to stimulate endothelium. Additionally, they are involved in the phagocytosis of complement-coated particles and immune complexes, as well as the regulation of leukocyte apoptosis [37]. Studies have demonstrated that missense variants in *ITGAM* impair the phagocytic function of monocytes, neutrophils, and macrophages. This impairment leads to the disrupted clearance of immune complexes, resulting in their deposition, tissue damage, and elevated levels of type I interferon (IFN-I) [38].

### 2.2. Genes of Innate Immunity

The second category encompasses genes involved in innate immunity and the associated signaling pathways, including IFN-I, Toll-like receptors (TLR), and nuclear factor κB (NFκB) [27]. These genes are grouped together due to their participation in innate immune responses.

IFN-I plays a vital role in the development of SLE, as demonstrated by the increased expression of IFN-I-inducible genes in the peripheral blood cells of the majority of SLE patients [39]. The significance of IFN-I-related genes in SLE susceptibility cannot be overstated, as more than half of the identified SLE susceptibility genes encode proteins directly or indirectly linked to IFN-I production or responses [27]. IFN-I exerts various functions and immune effects, including promoting the differentiation of monocytes and plasmacytoid dendritic cells, activating autoreactive T/B cells, stimulating autoantibody production, and inducing pro-inflammatory cytokines and chemokines [27]. Multiple triggers can induce IFN-I production in SLE, such as increased exposure of nucleic acids within immune complexes, necrotic debris, endosomal receptors (e.g., TLR7), or cytosolic sensors (e.g., IFIH1) [40].

TLR7 is located within intracellular endosomes and plays a central role in antiviral defense by recognizing single-stranded RNA. Activation of TLR7 can lead to both IFN-I production and NFκB activation in various cell populations, including dendritic cells, monocytes, macrophages, and B cells. IFN-I can contribute to the development of SLE [41]. It has been observed that sera from SLE patients contain TLR7 ligands in the form of immune complexes, which can activate plasmacytoid dendritic cells and induce IFN-I secretion. Furthermore, SLE sera can induce TLR7 expression in neutrophils, priming them for NETosis, which is also increased in SLE [42]. NETosis is a form of neutrophil cell death in which neutrophils release neutrophil extracellular traps (NETs) to capture and neutralize pathogens, thereby preventing their spread.

The *IFIH1* gene, also known as interferon-induced helicase-1, encodes the protein Mda5 (melanoma differentiation-associated protein 5), an intracellular receptor involved in recognizing double-stranded RNA. During viral replication, double-stranded RNA molecules bind to Mda5, initiating a cascade of events that result in the production of type I and III interferons (IFN-I and IFN-III) [43]. Gain-of-function mutations in IFIH1 lead to the activation of dendritic cells and macrophages, triggering the production of IFN-α in response to nucleic acids. This activation subsequently leads to T-cell activation and the production of autoantibodies [44].

The *IRF* (interferon regulatory factor) family of genes encodes proteins that regulate interferon transcription [45]. Three *IRF* genes, namely *IRF5*, *IRF7*, and *IRF8*, have been associated with SLE susceptibility. *IRF5* and *IRF7* are downstream proteins that interact with the MyD88 adaptor protein upon engagement of Toll-like receptors (TLRs), leading to the transcription of IFN-α mRNA. On the other hand, *IRF8*, which does not interact with MyD88, appears to be involved in the production of inflammatory cytokines in dendritic cells in response to TLR9 ligands [27,45]. Genetic variants in *IRF5* and *IRF7* associated with SLE susceptibility are considered gain-of-function variants and are related to increased serum IFN-α levels in SLE patients with specific autoantibodies [27,45]. However, no correlation was found between IRF5 and/or IRF7 and serum IFN-α levels in SLE patients without these autoantibodies [27,45].

*IRAK1*, situated on the Xq28 chromosome, encodes a serine-threonine protein kinase called IL-1 receptor-associated kinase 1, which plays a regulatory role in various pathways involved in both innate and adaptive immune responses. Its involvement in the regulation of NFκB and TLR activation, as well as the induction of IFN-α and IFN-γ, positions *IRAK1* as a promising candidate for thorough genetic and functional analysis in relation to SLE [46]. Jacob et al. propose that *IRAK1* may contribute to at least three immune cell functions that have been found to be abnormal in SLE: the induction of IFN-α and IFN-γ, regulation of the NFκB pathway, and TLR activation [46]. The identification of an X chromosome gene as a susceptibility factor in human SLE suggests that gender disparities in SLE might be influenced, at least in part, by sex chromosome genes.

The *TYK2* gene is situated on chromosome 19p13.2 and is responsible for encoding non-receptor tyrosine-protein kinase 2. *TYK2* belongs to the Janus kinase (JAK) family and plays a crucial role in the signaling pathways of IFN-I, IL-6, IL-10, IL-12, and IL-23, particularly in the transmission of signals from IFN-α and β. Recent research indicates that *TYK2* variants are associated with various autoimmune disorders, such as type 1 diabetes, psoriasis, multiple sclerosis, and increased susceptibility to SLE [47,48]. Interestingly, certain polymorphisms were found to have a protective effect in some autoimmune diseases while posing a risk factor for others, suggesting diverse underlying pathogenic mechanisms. In a study by Contreras-Cubas et al., genetic variants with a protective effect were identified for both childhood-onset and adult-onset SLE in the Mexican population [49].

The second group of SLE susceptibility genes includes genes associated with the NFκB pathway, such as *TNFAIP3*, *TNIP1*, *UBE2L3*, *PRKCB*, and *NFKBIA* [27]. The NFκB pathway regulates the activation of various cytokines, and NFκB target genes are involved in diverse immune functions, including lymphocyte development, activation, and differentiation, as well as the maturation and inflammatory functions of innate immune cells [50]. Abnormal NFκB signaling can lead to the production of autoreactive T-cells, which play a significant role in SLE, and promote plasma cell development.

The *TNFAIP3* (tumor necrosis factor alpha-induced protein 3) gene, which encodes the enzyme A20, has been demonstrated to inhibit NFκB activation, TNF-mediated apoptosis, and NLRP3 inflammasome [51]. Risk alleles of *TNFAIP3* are linked to reduced expression of A20 in SLE patients, resulting in heightened NFκB signaling.

TNIP1, also known as tumor necrosis factor alpha-induced protein 3-interacting protein 1, is a protein encoded by the *TNIP1* gene. It interacts with A20 and functions as a physiological inhibitor of NFκB. Variants of *TNIP1* that impair its inhibitory function contribute to the development of SLE by promoting increased NFκB activation [52].

Ube2l3, a ubiquitin-conjugating enzyme E2 L3, participates in the ubiquitination of NFκB precursor proteins to facilitate targeted degradation [50]. It may also play a role in B-cell proliferation and differentiation. The risk allele of Ube2l3 is associated with enhanced expression, leading to increased NFκB activation and elevated numbers of circulating plasma cells in SLE patients [53].

Prkcb, also known as protein kinase C beta type, is an enzyme involved in NFκB activation mediated by the B-cell receptor [50]. Variants of *PRKCB* have been linked to SLE, characterized by heightened NFκB activation and B-cell hyperactivity [54].

*NFKBIA*, or NFκB inhibitor alpha, is a transcription factor gene that participates in the activation of genes involved in immune responses [55]. Its association with SLE susceptibility is likely due to increased NFκB activation.

### 2.3. Genes of Adaptive Immunity

The third group of genes consists of those involved in adaptive immunity, specifically in the signaling and migration of immune cells. This group can be further divided into HLA genes and genes outside the HLA system. It encompasses various kinases, cytokines, and transcription factors associated with signal transduction within lymphocytes, as well as immune cell activation, proliferation, and interaction [27]. Variants in these genes may lead to the loss of immune cell tolerance and sustained production of autoantibodies.

The chromosomal region 6p21.3, referred to as the major histocompatibility complex (MHC) or human leukocyte antigen (HLA) region in humans, contains more than 200 genes. These genes encode leukocyte antigens, complement factors, and other molecules related to the immune system [33]. The MHC region is divided into three regions: class I, class II, and class III. Class I and class II regions consist of genes that encode glycoproteins responsible for processing and presenting peptides to T-cells. Class I molecules present peptides from within the cell to trigger CD8+ cytotoxic immune responses, while class II molecules present peptides from outside the cell to elicit helper T and B-cell antibody responses. The class III region encodes complement components, TNF, and other immune-related genes. Through meta-analysis of genome-wide association studies (GWAS), the HLA region has been identified as the most significant genetic risk factor for SLE [56,57]. However, the specific variants within this region that contribute to susceptibility are not yet fully understood due to its complexity. In white populations, the most consistent associations with SLE involve class II alleles *HLA-DR3* (DRB1*0301) and *HLA-DR2* (DRB1*1501) [58]. Large-scale GWAS studies have identified a combination of HLA alleles in class I (*B08:01* and *B18:01*), class II (DQB1*02:01, DRB3*02:00, and DQA*01:02), and an SNP (rs74290525) in *SLC44A4* in the class III region as the strongest associations [59]. Various mechanisms have been proposed to explain the association of DR and DQ alleles with autoimmunity, including variation in peptide binding regions, the selection of autoreactive T cells, and misfolded class II genes [60,61,62].

Apart from the HLA system, genes outside the HLA system, such as *PTPN22*, *BLK*, *BANK1*, *PXK*, *TNFSF4*, *ETS1*, *IKZF1*, *IKZF2*, *IKZF3*, *IL10*, and *BAFF*, also play roles in T- and B-cell signaling, transcription factors, and cytokines, and have been implicated in SLE susceptibility [27,63].

The gene *PTPN22* encodes the enzyme tyrosine phosphatase nonreceptor type 22, which is predominantly expressed in lymphoid tissues. This enzyme is involved in multiple signaling pathways associated with the immune response. Its main function is to inhibit T-cell activation and contribute to the central and peripheral tolerance of B-cells at various developmental stages [27,63,64]. A gain-of-function variant of *PTPN22* leads to the production of a more active phosphatase, resulting in lower thresholds for T-cell receptor signaling. This alteration affects B-cell receptor signaling, leading to increased autoreactivity and influencing the elimination of self-reactive B-cells during development. Ultimately, it contributes to both central and peripheral B-cell tolerance, thereby promoting autoimmunity [65].

The *BLK* gene encodes B-lymphoid tyrosine kinase (Blk), which plays various roles in intracellular signaling and regulates B-cell proliferation, differentiation, and tolerance [27]. Variants of the *BLK* gene associated with SLE susceptibility result in decreased expression of Blk, potentially affecting B-cell development and functional responses.

BANK1, encoded by the *BANK1* gene, is an adaptor protein known as the B-cell scaffold protein with ankyrin repeats 1. It facilitates the release of intracellular calcium and modifies the activation threshold of B cells [27]. Variants of the *BANK1* gene, linked to SLE, lead to reduced B-cell signaling and increased expansion of memory B-cells [66]. Rare variants found in patients impair the suppression of IFN-I in human B-cell lines and contribute to an increase in pathogenic lymphocytes in lupus-prone mice [67].

The *PXK* gene encodes a phox domain-containing protein involved in regulating synaptic transmission [27]. Risk variants in *PXK* associated with SLE lead to a decrease in B-cell receptor internalization. Although the genetic mechanism underlying this alteration is not fully understood, it may impact the regulation of B-cell signaling [68].

Tnfsf4, also known as tumor necrosis factor ligand superfamily member 4, is an inflammatory factor that has been associated with various inflammatory diseases and cancers. It is primarily expressed on activated CD4+ T cells. Increased expression of *TNFSF4* is believed to predispose individuals to SLE by promoting interactions between T-cells and antigen-presenting cells or by disrupting peripheral tolerance through the inhibition of IL-10-producing CD4+ type 1 regulatory T-cells [69].

ETS1, encoded by the *ETS1* gene, produces the protein C-ets-1, which belongs to the Erythroblast Transformation Specific family of transcription factors (ETS). Ets1 is primarily expressed in lymphocytes and is found at reduced levels in peripheral blood mononuclear cells from SLE patients [70]. It plays a crucial role in maintaining B-cell tolerance.

Ikzf1, Ikzf2, and Ikzf3 belong to the Ikaros family of zinc finger proteins. These proteins function as transcription factors and play a crucial role in regulating the differentiation, proliferation, and self-tolerance of lymphocytes. They are involved in controlling the signaling processes of B-cells, T-cells, and dendritic cells [71]. However, the specific mechanism by which causative variants in *IKZF1*, *IKZF2*, and *IKZF3* are associated with SLE is still unknown [27].

Interleukin 10 (IL-10) is an immunoregulatory cytokine with both immunosuppressive and immunostimulatory properties. It is primarily produced by B cells, which utilize it for proliferation, and myeloid cells, which employ it to suppress proinflammatory responses. In SLE patients, risk alleles of *IL10* lead to the increased production of IL-10 by peripheral blood B cells and monocytes, and elevated levels of IL-10 in the serum are correlated with disease activity [72]. Elevated levels of IL-10 contribute to SLE susceptibility and severity by promoting B-cell proliferation [73].

B cell-activating factor (BAFF) is a cytokine encoded by the T*NFSF13B* gene. It plays a significant role in the survival, proliferation, and maturation of B lymphocytes. Risk variants associated with BAFF increase its expression and are linked to active disease as well as renal and hematological involvement [74]. Excessive expression of BAFF is associated with enhanced survival and expansion of autoreactive B cells.

### 2.4. Genes with Unknown Immune Function

The fourth group comprises genes involved in immune functions, but their specific roles have not yet been fully elucidated. Some of these genes encode membrane proteins (e.g., *C3orf21*, *DHCR7*, *PLD2*), while others produce gene products with unknown immune functions [27].

The identified genetic variants or single nucleotide polymorphisms (SNPs) within the designated loci are common but have a relatively small effect on disease susceptibility, carrying a low relative risk of developing SLE. These variants explain approximately 30–50% of SLE heritability, indicating that other factors, such as rare genetic variants, epigenetic effects, and gene interactions (epistasis), play more significant roles in SLE susceptibility [75,76].

In addition to SNPs, copy number variations (CNVs) involving the deletion, insertion, or duplication of genomic regions also contribute to SLE susceptibility [77]. For example, the *NCF1* gene, which encodes neutrophil cytosol factor 1, is affected by certain SNPs that result in reduced oxidative burst and lower production of reactive oxygen species. This leads to increased expression of IFN-I-regulated genes and association with SLE [78]. Decreased CNVs (0 and 1 copy) of *NCF1* predispose individuals to SLE, while increased CNVs (≥3 copies) have a protective effect [79].

Complement components *C1q*, *C4A*, *C4B*, and *C2B* are the gene loci with the highest risk for developing SLE, followed by genes involved in the IFN-I signaling pathway (*IRF5*, *ITGAM*) and genes related to B lymphocyte signaling (*BANK1* and *BLK*).

Although SLE and other autoimmune diseases share many susceptibility loci, the role of a particular locus in predisposition is not always consistent across different diseases. Sometimes the same variant may have an opposite effect, or the degree of effect may vary. For example, certain *PTPN22* variants predispose to SLE but confer protection against inflammatory bowel diseases. Similarly, some *NCF1* variants show a strong association with SLE but only a mild association with rheumatoid arthritis and Sjögren’s syndrome [27].

SLE, a complex disease, is influenced by both genetic predisposition and environmental factors, highlighting its polygenic nature. These genetic variations often reside outside the coding segments of genes. To uncover common variants that may not reach genome-wide significance, it is crucial to focus on multiple independent variants within each locus, perform meta-analyses using available data, and promote international collaborations to strengthen association studies. Furthermore, integrating information from gene expression profiles, protein complexes, signal transduction pathways, and regulatory networks can offer additional insights into the disease [33].

## 3. Monogenic SLE

SLE has conventionally been regarded as a polygenic disease linked to gene polymorphisms. However, in rare cases comprising only 1 to 3% of all SLE patients, the disease can stem from a single gene mutation, giving rise to a form known as monogenic SLE [13,80]. While these forms are infrequent, they offer valuable insights into the mechanisms underlying the disease, enhancing our understanding of SLE’s pathogenesis and molecular mechanisms of immune tolerance, and facilitating the development of targeted treatment strategies [80]. The identification of monogenic lupus variants supports the idea that SLE is not a singular disorder but rather a heterogeneous collection of genetically distinct conditions, referred to as lupus-like diseases or lupus subtypes [33].

Several key characteristics raise suspicion of a monogenic form of SLE. These include an early onset of the disease, particularly before the age of five, evidence of Mendelian inheritance or a strong family history of the same disease, atypical clinical manifestations (such as severe cutaneous, neurological, or joint symptoms), resistance to standard therapies, male gender, and consanguinity, even in the absence of a positive family history [13,81].

Genome-wide association studies (GWAS) have identified several groups of genes involved in various physiological pathways as causative factors in monogenic SLE (Figure 1). Unlike polygenic SLE, monogenic forms are associated with nearly 30 genes that harbor single mutations in the coding regions of the genome [82]. Similar to susceptibility genes in polygenic SLE, genes responsible for monogenic forms can be categorized into several groups (Figure 1 and Table 2). These include genes associated with the complement system, such as deficiencies of complement components that play a crucial role in immune complex clearance. Another group comprises genes involved in lymphocyte signaling within T- and B-cells, as well as genes associated with IFN signaling pathways involving nucleic acid recognition or interferon production [27,83].

Hereditary deficiencies in specific complement components (C1q, C1r, C1s, C2, C3, C4A, C4B) have been linked to susceptibility to SLE [84]. Homozygous deficiency of the C1q component of complement, encoded by three genes (*C1QA*, *C1QB*, and *C1QC*) on chromosome 1, is associated with the highest prevalence of SLE, reaching up to 90% [85]. The risk associated with deficiencies in other complement components is lower. C1q deficiency leads to inadequate clearance of apoptotic debris, which can trigger the presentation of self-antigens and subsequent loss of tolerance. Monogenic SLE forms are characterized by early disease onset, recurrent pyogenic infections or infections caused by Neisseria meningitidis, frequent photosensitive skin rash, nephritis, oral ulceration, arthritis, and often the absence of antinuclear antibodies (ANA) [86,87]. C1r and C1s deficiencies are rare, and patients with these deficiencies typically succumb to severe infections at a young age [87]. Only 10% of patients with C2 deficiency develop SLE because the alternative complement pathway can bypass C2. These patients exhibit similar characteristics to other SLE patients but are more prone to infections. C4, encoded by C4A and C4B genes on chromosome 6, plays a role in increasing the number of self-reactive B-cells and altering B-cell tolerance. C4 deficiency is associated with the development of glomerulonephritis and high levels of autoantibodies [87]. Complement deficiencies are inherited as autosomal recessive disorders [87].

The subsequent group of monogenic SLE consists of mutations in genes associated with IFN signaling pathways, commonly known as interferonopathies. Examples include pathogenic variants in *TREX1*, *IFIH1*, *SAMHD1*, *RNASEH2A*, *RNASEH2B*, and *RNASEH2C*.

*TREX1* is a gene involved in DNA damage repair and is responsible for degrading genomic DNA in response to DNA damage. Mutations in *TREX1* lead to an excessive production of IFN-I due to the accumulation of self-DNA, as the clearance of extracellular, endosomal, and cytosolic DNA is compromised. This accumulated DNA acts as a dam-age-associated molecular pattern, inappropriately activating intracellular nucleic-acid-sensing pathways, triggering an IFN-I response and systemic inflammation [87,88]. Patients with *TREX1* mutations may develop various autoimmune diseases, including familial chilblain lupus, Aicardi–Goutières syndrome, retinal vasculopathy with cerebral leukodystrophy, and cerebral SLE. Familial chilblain lupus is characterized by painful, sometimes ulcerative, skin lesions resembling frostbite that appear in early childhood. Aicardi–Goutières syndrome presents as severe encephalopathy, progressive neurological damage, basal ganglia calcifications, white matter abnormalities of the brain, and, in some cases, skin changes resembling frostbite [89,90]. Most patients exhibit biallelic variants within *TREX1* with autosomal recessive inheritance, but some have been identified with heterozygous mutations and autosomal dominant inheritance [87].

*IFIH1* mutations in patients can result in early-onset SLE and Aicardi–Goutières syn-drome-like disease, including musculoskeletal involvement [87]. *IFIH1* mutations are inherited as autosomal dominant disorders [87].

The *SAMHD1* gene encodes the Sterile Alpha Motif (SAM) domain and Histidine-Aspartic (HD) domain-containing protein 1, which contributes to cellular stability and prevents reverse transcription of retroviruses [87]. *SAMHD1* mutations lead to increased DNA damage, subsequently upregulating IFN-stimulated genes. Patients with *SAMHD1* mutations can develop SLE, Aicardi–Goutières syndrome, and chilblain lupus. *SAMHD1* mutations are inherited as autosomal recessive disorders [87].

*RNASEH2A*, *RNASEH2B*, and *RNASEH2C* are three genes that encode the protein components of the RNaseH2 complex, an enzyme involved in breaking down RNA–DNA hybrids formed during DNA replication when they are no longer needed. Mutations in these genes cause an accumulation of ribonucleotides in genomic DNA during replication, leading to chronic DNA damage and IFN-I production [87]. Patients with these mutations exhibit cutaneous changes, photosensitivity, arthritis, lymphopenia, and autoantibody formation. *RNASEH2A*, *RNASEH2B*, and *RNASEH2C* mutations are inherited in a recessive manner [87].

Mutations within the third group of genes involved in lymphocyte signaling, particularly in T and B lymphocytes, can give rise to monogenic forms of SLE. It is important to note genes within the Ras/MAPK signaling pathway and PRKCD as significant contributors. The Ras/mitogen-activated protein kinase (MAPK) pathway plays a role in various cellular processes, such as proliferation, differentiation, and apoptosis, and is critical for T lymphocyte maturation in the immune system [87]. Genes associated with the Ras/MAPK pathway, including *PTPN11*, *KRAS*, *NRAS*, *SOS1*, *SHOC2*, and *SHP2*, are examples of mutations linked to the clinical presentation of “RASopathies” and are inherited in an autosomal dominant manner [87]. Mutations in these genes are associated with Noonan syndrome, characterized by facial dysmorphia, short stature, congenital heart defects, hemorrhagic diathesis, and an increased risk of malignancies. Some reported literature describes patients with SLE who also exhibit features such as hepatosplenomegaly, lymphadenopathy, an increased frequency of pericarditis, and autoimmune cytopenias [91]. Another example involves mutations in the *PRKCD* gene, which encodes protein kinase C delta (PKCδ), a protein involved in regulating B-cell development, proliferation, and apoptosis. Monogenic forms of SLE associated with *PRKCD* mutations result in dysregulated B-cell proliferation, loss of B-cell tolerance, hepatosplenomegaly, lymphadenopathy, and susceptibility to infections, particularly chronic Epstein–Barr virus (EBV) and cytomegalovirus (CMV) infections. These individuals exhibit typical features of SLE, including autoantibody production and an increased incidence of glomerulonephritis [88,92]. *PRKCD* mutations are inherited in an autosomal recessive manner [88].

Other instances of Mendelian inheritance in SLE involve mutations in the *DNASE1* (deoxyribonuclease 1) and D*NASE1L3* (deoxyribonuclease 1 like 3) genes, responsible for nucleic acid degradation. These enzymes play a role in digesting extracellular DNA from apoptotic cells. Dysfunction of these enzymes can lead to the activation of plasmacytoid and myeloid dendritic cells by circulating microparticles from apoptotic cells, resulting in the production of IFN-α [87]. *DNASE1* mutations are dominantly inherited, while *DNASE1L3* mutations are recessively inherited [87].

The *FASL* gene encodes the Fas ligand, which contributes to programmed cell death (apoptosis) [93]. Mutations in the *FASL* gene, inherited in an autosomal dominant manner, disrupt the removal of autoreactive cells and give rise to an autoimmune lymphoproliferative syndrome. These mutations have also been observed in patients with SLE [94].

Recently, mutations in the *LRBA* gene have been associated with cSLE [95]. The *LRBA* gene encodes lipopolysaccharide-responsive and beige-like anchor protein, an intracellular protein involved in regulating the trafficking of intracellular vesicles. *LRBA* promotes the expression of cytotoxic T lymphocyte-associated protein 4 (CTLA4). LRBA deficiency, inherited in an autosomal recessive manner, is associated with intense autoantibody production [96] and may contribute to the clinical manifestations of SLE [95].

## 4. Disparities in Gene Functions in Different Races in the Etiology of SLE

The prevalence of SLE is greater and its severity is higher among African American people and white Hispanic people compared to non-Hispanic white people [97]. Additionally, substantial evidence supports the existence of distinct susceptibility genes for SLE among African American people, white people, and Hispanic people [98]. Furthermore, lupus nephritis is a significant contributor to morbidity and mortality, with a higher prevalence observed in Asian populations compared to white populations [99]. When considering cSLE, the incidence is highest in female children of African descent and lowest in male children of white ethnicity [1].

However, it would be oversimplified and incorrect to attribute all disparities in health outcomes for SLE solely to genetic ancestry [100]. Racial and ethnic groups are diverse and heterogeneous, and their health conditions are influenced by numerous factors, including geographic location, socioeconomic status, educational attainment, and healthcare access, among others. To fully understand the root causes of health inequities and disparities, it is essential to consider the social and physical environment surrounding a particular population [101,102].

The literature reports varying outcomes between Asian and Caucasian populations, which can be attributed to the distinct genetic profiles of the study cohorts [103,104]. For instance, the association of the *PTPN22* gene with SLE susceptibility is well-established in Caucasian populations, but not in Japanese SLE cases. This discrepancy can be attributed to the significantly lower frequency of the *PTPN22* R620W polymorphism in the Japanese population compared to Caucasians [105]. Similarly, studies by Kyogoku et al. suggest that the *TYK2* SNPs associated with SLE in Caucasians do not confer a genetic risk in the Japanese population [103]. While homozygosity for the minor A allele of rs2304256 is slightly more prevalent in Japanese SLE patients than in healthy controls, a statistically significant association was not observed. The functional significance of *TYK2* suggests that it may be a significant risk factor for SLE in Caucasians, but a minor factor in Asians. Tang et al. conducted a study to investigate the association of several SNPs in the *IRF5* and *TYK2* genes, previously implicated in SLE susceptibility, with SLE in the Han Chinese population [104]. When comparing *IRF5* haplotypes among Japanese, Caucasian, and Han Chinese populations, Tang et al. observed distinct differences between Caucasian and Han Chinese populations. The Caucasian population risk haplotype, which includes the rs2070197 C allele, was not found in the Japanese and Han Chinese populations. Instead, a different risk haplotype [(exon6 (in)–rs10954213A–rs2004640T)] was identified in Han Chinese populations. Additionally, the authors demonstrated a significant association between *TYK2* rs2304256 and the development of SLE in the Han Chinese population [104]. This finding represents the first reported observation of a significant association between rs2304256 and SLE specifically in Han Chinese individuals.

According to a study of limited sample size, *HLA-DRB115:03* and *HLA-DRB108* were found to have higher frequencies in African American and Hispanic individuals with SLE, respectively [106]. Additionally, a candidate gene study focused on African American individuals identified *MECP2*, *MBL2*, and *PXK* as SLE susceptibility genes associated exclusively with individuals of European descent [107].

## 5. Association between Genetic Risk and Age of Onset in SLE

There is evidence suggesting that the genetic factors contributing to the development of SLE may vary between cSLE and adult-onset SLE cases. While candidate gene studies have not identified any genes specifically associated with cSLE, a study in a Korean cSLE population found unique SNPs: rs7460469 in *XKR6* and rs7300146 in *GLT1D1* [108]. Moreover, the relationship between *STAT4* and *SPP1* genes and cSLE has been validated in a Japanese population [109]. In the case of *TNFAIP3*, the genetic effect appears to be specific to males. In the case of polygenic diseases like SLE, it is generally accepted that assessing a genetic risk score provides a more comprehensive understanding of the genetic contribution to autoimmune diseases compared to investigating individual SNPs [110].

A study by Dominguez et al. explored the relationship between genetic risk and the age of SLE diagnosis, specifically examining the influence of HLA and non-HLA genetic risk scores [110]. The findings of this study, conducted on a multiethnic population, revealed distinct effects of non-HLA and HLA genetic risk scores on the age of SLE diagnosis. Higher non-HLA genetic risk scores were associated with a younger age of SLE diagnosis, indicating a stronger genetic influence. Conversely, higher HLA genetic risk scores were linked to an older age of SLE diagnosis. Overall, genetic risk scores accounted for 18% of the variation in the age of SLE onset [110].

In a similar study conducted by Webber et al., an association between known SLE risk loci and lupus nephritis risk was observed in both pediatric and adult populations with SLE [111]. The strongest effect was seen in European populations with cSLE, suggesting a more pronounced genetic influence on lupus nephritis risk in individuals with cSLE compared to those with adult-onset SLE, supporting the hypothesis of a greater genetic role in the development of SLE at younger ages [111].

## 6. Epigenetics in SLE

Epigenetic dysregulation plays a significant role in the development of SLE [112]. Epigenetic changes refer to functional modifications in the genome that do not involve alterations in the DNA sequence but impact gene activity and expression, potentially leading to heritable phenotypic changes. The pathogenesis of SLE involves three primary epigenetic mechanisms: changes in DNA methylation, histone modifications, and noncoding RNAs (ncRNAs) within autoreactive T-cells and B-cells [113].

DNA methylation is a process where methyl groups are added to the DNA molecule, typically resulting in the suppression of gene transcription. SLE patients exhibit global T-cell hypomethylation, leading to the overexpression of genes related to autoimmunity [114]. A genome-wide DNA methylation study identified specific sites within the promoter regions of 14,495 genes that were hypermethylated (105 sites) or hypomethylated (236 sites) in CD4+ T-cells of SLE patients compared to healthy controls [115]. The degree of hypomethylation of CG dinucleotides correlates with autoantibody production, anti-dsDNA level, and disease activity [113]. In women, demethylation of the X chromosome may contribute to the higher prevalence of SLE among females [113].

Histone modifications, involving acetylation and methylation of the proteins that package DNA into nucleosomes, also play a crucial role in SLE. Acetylation adds an acetyl group to histone proteins, resulting in a transcriptionally active chromatin structure (euchromatin) that enhances gene expression. Conversely, deacetylation leads to an inactive, condensed chromatin structure (heterochromatin). Histone methylation involves the transfer of methyl groups to histones, and its effects on gene expression can either activate or repress transcription. Histones form octamers composed of two copies each of histones H2A, H2B, H3, and H4. In CD4+ T-cells of SLE patients, histones H3 and H4 are hypoacetylated, and histone H3K9 is hypomethylated [116]. Additionally, neutrophil extracellular traps (NETs) from SLE patients contain higher levels of acetylated H4-K8,12,16 and H2B-K12. The hyperacetylated chromatin of NETs may activate myeloid and plasmacytoid dendritic cells and trigger the activation of autoreactive T- and B-cells [116].

MicroRNAs (miRNAs) are small single-stranded ncRNA molecules that play crucial roles in RNA silencing and post-transcriptional regulation of gene expression. They inhibit the translation of target genes and/or reduce the stability of messenger RNA (mRNA). The dysregulation of certain ncRNAs is another epigenetic mechanism involved in SLE pathogenesis. For instance, a reduced expression of microRNA-146a (miR-146a) was observed in peripheral blood mononuclear cells (PBMCs) from SLE patients [117]. miR-146a acts as a negative regulator, preventing excessive activation of inflammatory responses in multiple immunological pathways, including the IFN-I pathway. Decreased miR-146a expression leads to the upregulated expression of IFN response genes in SLE patients [117].

## 7. Environmental Triggers in SLE

Multiple environmental factors, including ultraviolet (UV) light, particularly UVB, infections, toxins, and certain medications, are believed to have a role in triggering and worsening SLE [10]. Some of these environmental triggers may exert their influence through epigenetic mechanisms. For example, exposure to UV light is thought to induce apoptosis of keratinocytes, leading to the release of DNA degradation products on the cell surface. These DNA fragments can act as triggers, stimulating the production of antibodies that target components of the nucleus [10]. Additionally, there are hypotheses suggesting that infections, particularly herpesviruses like Epstein–Barr virus (EBV), may activate the innate immune system and promote the differentiation of B lymphocytes, thereby initiating the autoimmune process and stimulating the production of autoantibodies. However, the intricate details of these complex mechanisms are not yet fully understood. Certain medications, such as minocycline, procainamide, chlorpromazine, and interferon alpha, have been associated with the development of SLE due to their impact on patterns of DNA methylation. Smoking, known to induce an inflammatory response, is also recognized as a risk factor for SLE. Furthermore, early-life risk factors, including low birth weight (<2500 g), preterm birth (≥1 month early), and exposure to agricultural pesticides, have been suggested to contribute to the development of SLE [118].

## 8. Hypothetical Model of SLE Development

**Figure 2:**
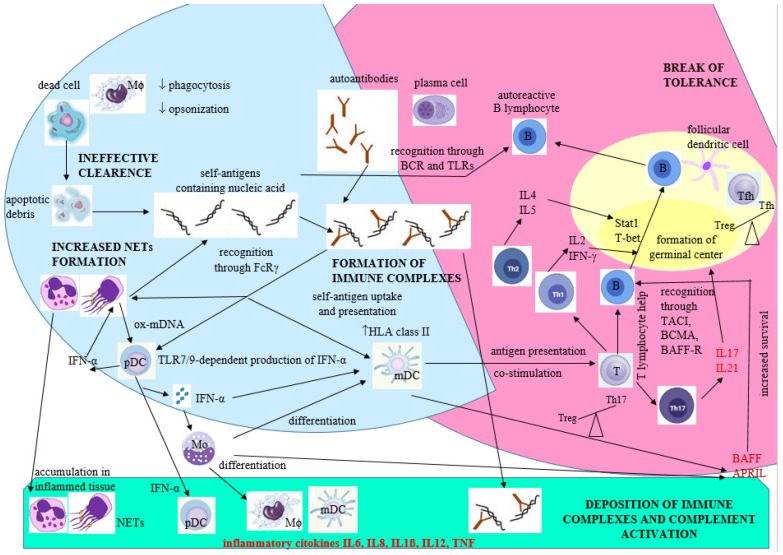
Hypothetical model of polygenic SLE development: adaptive immune disorders lead to the generation of autoantibodies, specifically antibodies targeting self-antigens (right side of the figure, marked in pink). These autoantibodies progressively accumulate as a consequence of the innate immune dysfunction (left side of the figure, marked in blue). Modified according to reference [121]. APRIL: a proliferation-inducing ligand; B: B-cell; BAFF: B-cell-activating factor; BAFF-R: B-cell-activating factor receptor; BCMA: B-cell maturation antigen; BCR: B-cell antigen receptor; FcRγ: Fc receptor-γ; HLA class II: human leucocyte antigen class II; mDC: myeloid dendritic cell; Mϕ: macrophage; NET: neutrophil extracellular trap; ox-mDNA: oxidized mitochondrial DNA; pDC: plasmacytoid dendritic cell; Stat1: signal transducer and activator of transcription (a transcription factor); T: T-cell; TACI: transmembrane activator, calcium modulator and cyclophilin ligand interactor; T-bet: a T-box transcription factor; Tfh: T follicular helper; TLR7/9: Toll-like receptors 7 and 9.

According to certain hypotheses, the progression of the disease can be divided into three phases [119]. In the initial phase, which is asymptomatic, the disruption of immune tolerance to nuclear self-antigens occurs due to the interplay of environmental, genetic, and epigenetic factors, although the exact mechanisms are not fully understood. The second phase involves further dysregulation and amplification of the compromised immune response, which can be observed by the detection and measurement of various antibodies like ANA in laboratory tests. The final phase, known as the third phase, is characterized by inflammatory reactions leading to damage in target organs (such as the skin, kidneys, blood vessels, joints, brain, etc.), thereby manifesting the clinical symptoms of the disease [10]. SLE affects both innate and adaptive immunity, resulting in immune system disorders [120,121] (Figure 2).

**Figure 3:**
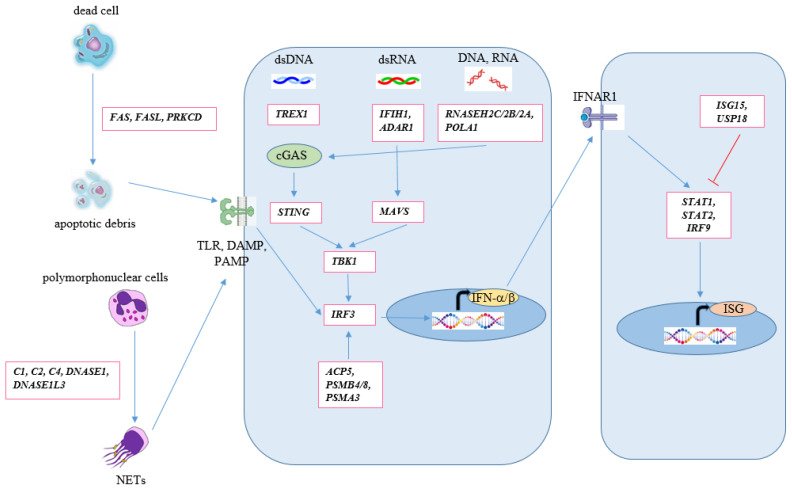
Pathways included in monogenic SLE development. The accumulation of nuclear material in the extracellular space resulting from apoptosis and NETosis triggers Toll-like receptors (TLRs). These TLRs, along with other pathways impacting the crucial transcription factor IRF3, are involved in nucleic acid recognition and degradation. Impairment in nucleic acid sensing or compromised handling of nucleic acid-containing waste products leads to a type I interferon response. This interferon response activates a set of interferon-stimulated genes. Genes are indicated within boxes. Modified according to references [122,123]. cGAS: cyclic GMP–AMP synthase; DAMP: damage-associated molecular patterns; IFNAR1: interferon alpha and beta receptor subunit 1; NET: neutrophil extracellular trap; PAMP: pathogen-associated molecular patterns; TLR: Toll-like receptors.

Innate immune disorders contribute to increased exposure to self-antigens and involve reduced clearance of apoptotic cells, diminished phagocytosis, and heightened formation of neutrophil extracellular traps (NETs) through a process called NETosis. These NETs consist of neutrophilic DNA, RNA, and histones, which have immunogenic properties. In SLE patients, these extracellular traps are not effectively degraded in the bloodstream. Plasmacytoid dendritic cells respond to these factors by releasing IFN-I. Furthermore, oxidized mitochondrial DNA released by neutrophils in SLE can stimulate plasmacytoid dendritic cells to produce IFN-I. IFN-I promotes the differentiation of monocytes into myeloid dendritic cells, augmenting their antigen-presenting capabilities, including the presentation of autoantigens to T lymphocytes. T lymphocytes, in turn, produce various cytokines and molecules that intensify the immune response against self-antigens and contribute to inflammation. The breakdown of immune tolerance eventually leads to an increase in autoreactive effector B lymphocytes. B lymphocytes are stimulated by T lymphocytes through interactions involving CD40 on B lymphocytes and CD40 ligand on T lymphocytes. TNF produced by dendritic cells, BAFF secreted by myeloid cells, APRIL expressed by T-cells, dendritic cells, monocytes, and macrophages, exposure to self-antigens, T-cell cytokines, and other factors play crucial roles in stimulating B lymphocytes to generate autoantibodies. Signal transducer and activator of transcription 1 and T-box transcription factor contribute to the production of pathogenic autoantibodies. Follicular dendritic cells also play a critical role in the activation and selection of B-cells within germinal centers in secondary lymphoid organs. Ultimately, B lymphocytes produce antibodies that target self-antigens, forming immune complexes that deposit in tissues. These immune complexes activate the complement system, recruit myeloid cells (especially neutrophils), and induce the release of enzymes from neutrophil granules and reactive oxygen radicals from macrophages, resulting in inflammation and damage to target organs. Immune complexes can be taken up by B-cells through the B-cell antigen receptor or by dendritic cells through Fc receptor-γ, activating intracellular innate receptors like TLR7 and TLR9, which subsequently produce inflammatory cytokines, including IFN-I (Figure 2 and Figure 3) [122,123].

In summary, due to the dysfunction of the innate immune system, adaptive immune disorders arise, resulting in the production of autoantibodies that specifically target self-antigens. These autoantibodies gradually accumulate as a consequence of the impaired innate immune function.

## Figures and Tables

**Table 1:** Differences in clinical manifestations between childhood-onset systemic lupus erythematosus (cSLE) and adult-onset systemic lupus erythematosus (SLE). The table is based on references [5,8,9,10].

	cSLE	SLE
**Gender difference**	4–5 girls to 1 boy	9 females to 1 male
**Severity of clinical picture**	More severe, often affects multiple organs and systems	Compared to children, the disease in adults is usually less active at the time of diagnosis
**Renal involvement**	60–80%	35–50%
**Central nervous system involvement**	20–50%	10–25%
**Pulmonary involvement**	15–40%	20–90%
**Joint involvement**	60–70%	80–95%
**Treatment**	More intensive, glucocorticoids and immunosuppressants more frequently used	Compared to children, glucocorticoids and immunosuppressants less frequently used
**Specific complications**	Poor growth, delayed puberty, higher risk of corticosteroid-related complications	Malignancy
**Examples of variants distinct between cSLE and SLE**	*ESR1* *ORα* polymorphisms *MBL2* rs7460469 in *XKR6* rs7300146 in *GLT1D1* *STAT4* *SPP1* *TNFAIP3*	*ESR2* *ORα* polymorphisms *MECP2* *PDCD1*

**Table 2:** The most important genes responsible for monogenic forms of SLE.

Type of Gene Function Disorder	Gene	Inheritance	Clinical Picture
**Clearance defects**	Hereditary deficiencies in specific complement components (*C1QA*, *C1QB*, *C1QC*, *C1R*, *C1S*, *C2*, *C4A*, *C4B*)	AR	Early disease onset, recurrent pyogenic infections or infections caused by *Neisseria meningitidis*, frequent photosensitive skin rash, nephritis, oral ulceration, arthritis, and often the absence of antinuclear antibodies
**IFN signaling pathways**	*TREX1*	AR/AD	Familial chilblain lupus, Aicardi-Goutières syndrome, retinal vasculopathy with cerebral leukodystrophy, and cerebral SLE
	*IFIH1*	AD	Early-onset SLE and Aicardi-Goutières syndrome-like disease, including musculoskeletal involvement
	*SAMHD1*	AR	Aicardi-Goutières syndrome, chilblain lupus, SLE
	*RNASEH2A*, *RNASEH2B*, *RNASEH2C*	AR	Cutaneous changes, photosensitivity, arthritis, lymphopenia, and autoantibody formation
	*DNASE1*	AD	SLE, high titers of autoantibodies
	*DNASE1L3*	AR	SLE, very early onset, frequent glomerulonephritis
**Lymphocyte signaling**	*PRKCD*	AR	Hepatosplenomegaly, lymphadenopathy, and susceptibility to infections, particularly chronic EBV and CMV, autoantibody production and an increased incidence of glomerulonephritis
	*PTPN11*, *KRAS*, *NRAS*, *SOS1*, *SHOC2*, *SHP2*	AD	Noonan syndrome, hepatosplenomegaly, lymphadenopathy, an increased frequency of pericarditis, and autoimmune cytopenias
	*FASL*	AD	SLE with lymphadenopathy, autoimmune lymphoproliferative syndrome

## Data Availability

Not applicable.
